# Assessing the validity of the clinician-rated distress thermometer in palliative care

**DOI:** 10.1186/s12904-019-0466-x

**Published:** 2019-10-17

**Authors:** A. Van Lander, A. Tarot, C. Savanovitch, B. Pereira, B. Vennat, V. Guastella

**Affiliations:** 1 CHU Clermont-Ferrand, Unit of palliative care, Hôpital Louise Michel, 63118 cébazat, France; 2 University Clermont Auvergne, ACCePPT, Place Henri Dunand, 63000 Clermont-Ferrand, France; 3 CHU Clermont-Ferrand, DRCI, 63000 Clermont-Ferrand, France

**Keywords:** Distress, Palliative care, Clinician-rated, Distress thermometer

## Abstract

**Background:**

The distress of patients suffering from a terminal illness can lead to a state of despair and requests for euthanasia and assisted suicide. It is a major challenge for palliative care workers. The Distress Thermometer (DT) is recommended by the National Comprehensive Cancer Network as a means of more easily assessing distress. It is available as a Self-assessment reported Distress Thermometer, but for a wider use in palliative care it should also be implemented in the form of a clinician-reported outcome (clinRO). Clinicians need to rate patient’s distress when the patient is not able to do so (subject that cannot be addressed, defensive patient…). The primary aim of the quantitative study was to assess the validity of the Clinician-Rated Distress Thermometer in palliative care.

**Method:**

The assessments were performed by teams working in three palliative care centres. The primary endpoint was concordance between the patient and clinicians’ responses via Lin’s concordance coefficient. Eligible patients were aged 18 years or older, suffering from a severe disease in the palliative phase, and with a sufficient level of awareness to consent to participate in the study. A total of 51 patients were recruited, 55% were male, with a mean age of 65.8 years [39–90 years].

**Results:**

Three hundred sixty-four clinician-Rated Distress Thermometer and 467 Self-Reported Distress Thermometer were performed. Only 364 of the 467 Self-Reported Distress Thermometer were used for the study, as investigators did not systematically ask the patient to give an account of his distress. Concordance between patient and clinician responses: The Lin’s concordance coefficient with a threshold (alpha) of 5% was 0.46 [0.38; 0.54]. At the first assessment, it was 0.61 [0.44; 0.79]. The Cohen’s kappa coefficient was 0.52, with a concordance rate of 79.6%. The sensitivity was 82.9% [66.4–93.4] and the specificity 71.4% [41.9–91.6].

**Conclusion:**

The first assessment gave the best results in terms of concordance between Clinician-Rated DT and Self-Reported DT. In the next assessments, the Clinician-Rated DT were less consistent with the patients’ Self-Reported DT.

## Background

Since the work of Jimmie Holland’s interdisciplinary group [1], the concept of psychological distress applied to patients suffering from a severe disease has been widely developed. It is described as an “unpleasant experience of an emotional, psychological or spiritual nature that interferes with the ability to manage one’s treatment” and which “ranges in a continuum from a normal common feeling of vulnerability, sadness and fear to more disabling conditions such as anxiety, panic attacks, depression and spiritual crisis”. The concept does not characterize the patients as requiring psychiatric care but identifies the need for a specific management approach [2]. The National Comprehensive Cancer Network (NCCN) asserts that distress should be systematically assessed to tailor care to the individual. One simple means of assessment is the Distress Thermometer (DT), a self-report measure consisting of a line with a 0–10 scale going from “No distress” to “Extreme distress”. Questions on the Problem List concern the previous week and the assessment day itself. The threshold of significance varies between 4/10 and 5/10 with a sensitivity ranging from 0.77 to 0.80 and a specificity from 0.59 to 0.70 (3, 4). At least one third of patients suffering from cancer present a significative distress [3–5]. Studies have shown that the DT, in comparison with other screening tools such as the Hospital Anxiety and Depression Scale (HADS), has a good validity and is easy to use [5, 6]. The assessment of distress is also essential in palliative care. In August 2017, the WHO stated that palliative care “prevents and alleviates suffering by the early identification, accurate assessment and treatment of pain and other problems, be they physical, psychosocial or spiritual” [7]. “Management of suffering involves addressing problems other than physical symptoms” [8]. The DT (a French version of the DT was validated in 2008) could play a useful role in this process [6]. Its threshold of significance is 3/10 with a sensitivity of 0.76 and specificity of 0.82. A study made in a French palliative care unit [9] reported that the DT identified extreme distress in 42% of the patients investigated. One limitation of the DT is its use as a Self-Report tool, which requires health professionals to be able to question patients on their psychological condition. One study in which the DT was used as a Clinician-Rated Distress Thermometer [10] was carried out by psychologists on a sample of 340 patients: for one year, 14 psychologists analysed their accompaniments using a booklet. To check the results, a second group of 12 psychologists throughout France reiterated the experience. Statistical analysis was performed with STATA 10.0 and the free part with Alceste. 801 interviews conducted among 237 patients aged 67 years (33–95) demonstrated that the lethal disease generates an identity crisis which is experienced by the patients with a feeling of distress. The aim of the present work was to examine the validation and reliability of the DT in a clinician-reported outcome (ClinRO) screening taking into account the profession of the assessors and the duration of care.

## Methods

### Participants

Eligible participants aged 18 years or older were recruited over a 6-month period in 2017. Inclusion criteria were: receiving palliative care for a severe disease, ability to reply to simple questions (score greater than 10 on the Glasgow scale and scores of 1 or 2 on the Rudkin scale) and consent to participate in the study. To avoid recruitment bias, three palliative care teams took part in the study:

- A palliative care unit (PCU), representative at the national level, in which mean patient stay is 11 days, having an active queue of 200 patients (2016) and dealing with diverse diseases including cancer, blood disorders, neurological disorders and organ failure.
- A cancer outpatient department (COD) working with palliative care patients undergoing chemotherapy.
- A regional ambulatory palliative care team.

### Procedure

The patients received all the information required, data privacy and anonymity were protected. The assessments were performed by 8 physicians, 20 nurses and auxiliary nurses and 3 psychologists. The study was approved by the local ethics committee (IRB du CPP Sud-Est VI) in February 2016 as part of the ongoing NUAncE research project (n°2016/CE17). Health professionals (physicians and nurses) and psychologists completed the DT, giving their estimation of the patient’s distress before they did so.

### Data collected and measures

In order to figure out the very validity of the Clinician-Rated DT, it was used on patients who were likely to encounter some distress during the period of medical care (on the basis of the self-statements). Sensitivity alongside specificity of a test give an evaluation of its intrinsic validity [11]. Statistically, the sensitivity of a test measures its ability to give a positive result when a hypothesis has been verified. It comes in opposition to specificity which measures the ability to give a negative result when the hypothesis has not been verified. All results concerning the evaluation of sensitivity were those modeled, using statistical approaches for repeated data (generalized estimating equations).

The DT was first used by the health professionals by observing the patient and by empathy. Their assessments were not influenced by those of the patients. Then the patients used the DT to assess their own distress. The PCU made assessments every day and the two other teams once a week. The results were entered into a database. Focus groups were created from members of the different teams to collect feedback on their use of the DT: focus groups consisted in approximately 15 investigators per site, doctors and psychologists, who met during three reunions.

### Statistical analysis

Sample size estimation was determined according to COSMIN recommendations (http://www.cosmin.nl/) to validate the Clinician-Rated DT screening.

Statistical analysis was performed with Stata 13 (StataCorp, College Station, TX). The tests were two-sided with a type-I error set at 5%. Continuous data were presented as mean ± standard-deviation or median [interquartile range], according to statistical distribution [12]. The assumption of normality was studied by the Shapiro-Wilk test. The concordance between the assessments of the health professionals and those of the patients was calculated by comparing DT scores from 0 (No distress) to 10 (Extreme distress) according to Lin’s concordance coefficient for repeated data. The concordance between professional and patient assessment of significant distress (threshold of 3/10) was analyzed by Cohen’s kappa coefficient and concordance rate. When appropriate (assessment as statistic unit), estimates of sensitivity and specificity were obtained by first estimating the sensitivity and specificity across all health professionals for each patient. Sensitivity and specificity were then estimated by calculating the average of the individual specific estimates across patients. The variance of the estimate is the sample variance divided by the number of patients. Generalized estimating equations with logit link and working independence correlation structure were also used to estimate sensitivity, taking into account the correlation among the multiple measures for the same patient (owing to assessment by three different categories of health professionals, physicians, nurses and psychologists). Lin’s coefficient, sensitivity and specificity were presented with 95% confidence intervals whereas kappa values were studied according to the usual recommendations (11): < 0.2 (negligible), 0.2–0.4 (low/weak consistency), 0.4 to 0.6 (moderate agreement), 0.6–0.8 (substantial/good agreement) and > 0.8 (excellent agreement).

## Results

### Demographic and clinical characteristics (Table 1, Fig. 1)

A total of 51 patients (Table 1) were included in the study, 55% of them were male, with a mean age of 65.9 +/− 11.4 years [39–90]. Of the 51, 36 were hospitalized in the PCU, 9 were cancer outpatients and 6 were in ambulatory care. Twenty-three patients (45.1%) were married, 10 (19.6%) were divorced and a further 10 were widowed, 5 (9.8%) were living with a partner and 3 (5.9%) were single. Most (70.8%) of the patients were retired. Of the 29.2% who had an occupation, the largest proportion (25%) were salaried employees. The patient’s relatives or friends were present in their immediate environment in almost all cases (96.1%). The main disease involved was cancer (86.3%), followed by blood disorders (7.9%) and neurological disorders such as amyotrophic lateral sclerosis (ALS) (5.9%). Thirty nine patients (88.6%) had metastases, 22 (52.9%) to the lungs, 17 (44.7%) to the liver, 16 (42.1%) to the bones, 15 (39.5%) to the lymph nodes, 10 (26.3%) to the peritoneum and 9 (23.7%) to the brain. All the patients recruited were informed of their diagnosis and prognosis. Five (10.2%) patients had a history of mood disorder [13].

**Fig. 1:**
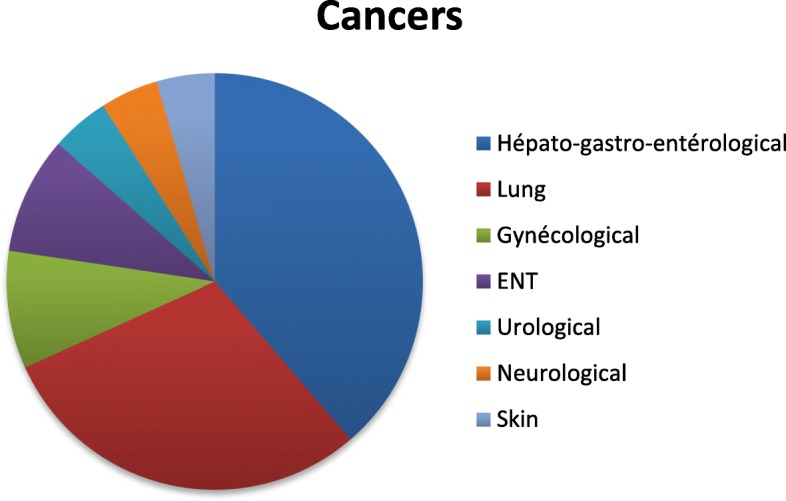
Cancers

**Table 1:** Demographic and clinical characteristics

		Number	Rate (%)
Sociodemographic characteristics			
Palliative Care Unit		36	70
Cancer Outpatient Department		9	18
Ambulatory palliative care team		6	12
Men		28	55
Age	Average (years)	65,8	
Family Status	With partner	28	55
Professional Status	Active	13	25
Professional status	Retired	34	67
Presence of social support	Yes	49	96
Disease-related characteristics			
Disease	Cancer	44	86
Metastatic Cancer	Yes	39	88
Time between diagnosis of disease and death	Mean (months)	28.8	
Psychological characteristics			
Psychiatric disease	Yes	5	10
Dementia	Yes	1	2

### DT score

Of the 467 Clinician-Rated DT performed, which represents a median number of 9.27 +/− 10.3 assessments per patient, 437 (93.6%) were made in the PCU, 17 (3.6%) in ambulatory care and 13 (2.8%) in the COD. The first assessment was made 25.1 months ±28.4 [IQR: 12.4 [6.1; 38.1]], after diagnosis and 25.5 days (+/− 30.2) [IQR: 9[1; 29]] before death. Time between the first and last assessment was 17.19 days (+/− 20.3), and time between the last assessment and death 8.6 (+/− 17.8) days [IQR: 4[2; 7]]. The Clinician-Rated DT also has an acceptable retest reliability.

**Fig. 2:**
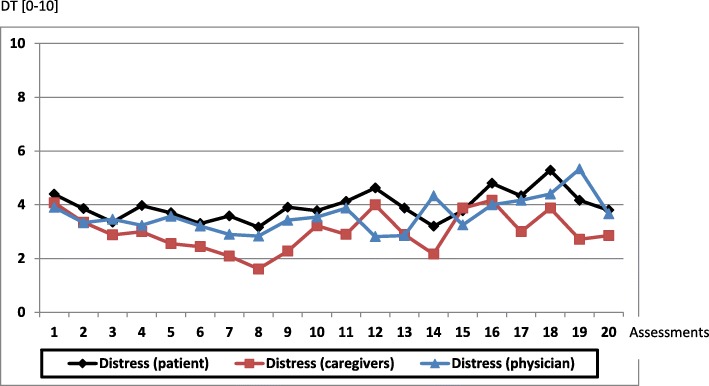
Results of DT

A total of 364 Self-Reported DT were made by the patients, 80.4% of them recorded significant distress at least once. Only 20% of the Self-Reported DT had a score of 0/10 (No distress). The mean DT score was 3.8/10 +/− 2.8. The mean distress score on the corresponding Self-Reported scale was 3.4/10 +/− 2.6. In both assessment modes, the highest distress score was that recorded at the first evaluation (Fig. 2).

### Relationship between patient and clinician assessments

The Lin’s coefficient concordance between the 364 Self-Reported DT and the 364 Clinician-Rated DT was 0.47 [0.39; 0.54]. The first assessments recorded the best results in terms of concordance: Lin’s coefficient concordance between the 49 Self-Reported DT and the 49 Clinician-Rated DT was 0.62 [0.44; 0.79].

The Cohen’s kappa coefficient associated to the comparison of the 364 Self-Reported DT and the 364 Clinician-Rated DT was 0.33 with a concordance rate of 67.3%. The sensitivity was 66.8% [60.3–72.9] and the specificity 68.1% [59.6–75.9]. Again, the results were better for assessments made at the first evaluation. Cohen’s kappa coefficient was 0.52 with a concordance rate of 79.6%. Sensitivity was 82.9% [66.4–93.4] and specificity 71.4% [41.9–91.6].

### Relationship between patient and clinician responses according to the different health professionals

The nurses performed 222 assessments. The Lin’s coefficient concordance was 0.54 [0.45–0.63]. The Cohen’s kappa coefficient was 0.37 with a concordance rate of 68.5%. The sensitivity was 65.5% [56.9–73.3] and the specificity 73.5% [62.7–73.3]. In contradiction with the self-assessments, the healthcare providers rated the patients on 22 occasions as being in distress and on 48 occasions as not being in distress. Concordance was best achieved during the first evaluation with Lin’s coefficient equals being 0.75 (*p* < 0.001). The kappa coefficient was 0.84 with a concordance rate of 92.9%, with a sensitivity of 100% [66.4–100] and a specificity of 80% [28.4–99.5]**.** Out of 14 evaluations, the nurses made only one mistaken evaluation, wrongly considering that the patient was in distress. During the second assessment, the Lin’s coefficient concordance coefficient was of 0.60 (*p* < 0.001). The kappa coefficient was 0.42 with a concordance rate of 71.4%, a sensitivity of 66.7% [29.9–92.5] and a specificity of 80% [28.4–99.5]. The healthcare providers wrongly rated a patient as being in distress in 4 of the 14 assessments and underestimated distress on three other occasions.

The physicians performed 126 assessments. The Lin’s coefficient concordance was 0.26 (*p* < 0.001) and the kappa at 0.24, with a concordance rate of 64.3%, a sensitivity was of 67.9% [56.8–77.6] and a specificity 57.1% [41–72.3]. In 126 assessments, the physicians’ assessments were not consistent with the patients on 45 occasions, 18 times considering that the patients were suffering from distress and 27 times that they were not. Concordance was the best during the first evaluation and decreased afterwards: the Lin’s coefficient concordance was 0.42 (*p* = 0.009), the Cohen’s kappa coefficient was 0.32, with a concordance rate of 73.1%, a sensitivity of 72.7% [49.8–89.3] and a specificity of 75% [19.4–99.4]. The physicians were at variance with the patients in 7 of the first 26 assessments, considering one patient to be in distress and 6 others as not being in distress. At the second evaluation, the Lin’s coefficient concordance was of 0.31 (*p* = 0.307). The kappa coefficient was of 0.21 with a concordance rate of 70%, a sensitivity of 85.7% [42.1–99.6] and a specificity of 33.3% [0.8–90.6]. The physicians made errors in 3 out of the 10 assessments, mistakenly identifying distress in 2 patients and failing to identify distress in another one.

The psychologists performed 16 assessments overall. The Lin’s coefficient concordance was of 0.75 (*p* < 0.001). The kappa coefficient was of 0.50 with a concordance rate of 75%, a sensitivity of 83.3% [35.9–99.6] and a specificity of 70% [34.8–93.3]. The psychologists made errors in 4 out of the 16 assessments mistakenly identifying distress in 3 patients and failing to identify distress in another one.

### Focus group feedback

The health professionals expressed their difficulties in using the DT as a self-assessment tool: “The word distress is a strong term. Won’t I be running the risk of making things worse or setting something off if I ask a patient about her or his distress? ” “I couldn’t see myself talking about distress. I could see he was obviously in distress but we couldn’t do anything for him. If we bring up the subject what are we going to do about it?” They tended to suggest drugs for mood disorder such as anxiolytics and were reluctant to accept a refusal: “What’s the use of assessing distress if we can’t treat it?” “Is it worth asking these questions?” The psychologists did not express these misgivings.

## Discussion

The statistical power of the study was good with 467 assessments performed. The profile of the patients included was satisfactory since it corresponded to that of patients in two other French studies of distress in a palliative care setting [9, 10].

### Limits

The inclusion rate in the PCU was low (36%) in proportion to the number of patients hospitalized, but it is consistent with the inclusion rates used in other studies [14]. The exclusion criteria were impaired with alertness and awareness. On admission to the hospital, the patients were already in the terminal phase of their illness and their stay in the unit was accordingly short (less than 15 days). To have a better representativeness, we included two other patient categories but the inclusion rate was again low and recruitment was slower. The different teams had scant experience of research activity and needed to attend explanatory meetings. The difficulties experienced by the nurses in questioning the patients limited the number of self-assessments. We did not know what training the professionals had received, nor their age or seniority. These elements can influence their evaluation. A study with a larger number of professionals is needed for the results to be fully reliable.

### External validity

The DT had a good external validity as a clinician-reported outcome (ClinRO). The mean of the assessments (3.8/10) was higher than in the French study (2.9/10) [6], which, however, was performed in a cancer ward and not in a PCU, but was closely consistent with results of studies performed in other PCUs: 3.9/10 of Van Lander’s study [10] and 4.4/10 in that of Thekkumpurath. The longitudinal evolution, “The longitudinal course of the interviews shows a possible identity transformation and distress reduction” [10] (Table 2), was also similar to that of Van Lander’s study.

**Table 2:** Relationship between Clinician-Rated Distress Thermometer and Self-Reported Distress Thermometer according to profession

	Kappa/Agreement rate (%)	Sensitivity (IC95)	Specificity (IC95)
Health professionals			
All	0.33/67	67 [60.3–72.9]	68 [59.6–75.9]
1st evaluation	0.52/80	83 [66.4–93.4]	71 [41.9–91.6]
Nurses			
All evaluations	0.37/68	65 [ 56.9–73.3]	73 [62.7–73.3]
1st evaluation	0.84/93	100 [66.4–100]	80 [28.4–99.5]
Physicians			
All evaluations	0.24/64	68 [56.8–77.6]	57 [41–72.3]
1st evaluation	0.32/73	73 [49.8–89.3]	75 [19.4–99.4]
Psychologists			
All evaluations	0.50/75	83 [35.9–99.6]	70 [34.8–93.3]

### Internal validity

As described in the Statistical Section, generalized estimating equations with logit link and working independence correlation structure were used to estimate the sensitivity, taking into account the correlation between the multiple measures for the same patient. When appropriate the delay between the first and the next assessments was taken into account as a covariate, just like the number of assessments. Furthermore, the intra-center correlation coefficient for Clinician-Rated DT was low at 5.5%, whereas the intra-patient correlation coefficient (taken into account in our analyses) was equal to 51%. Considering the center effect (as random-effect), the results were not modified, and not impacted by the imbalance sample size of the three sites.

The internal validity was good with a satisfactory Kappa in the first three assessments. Then the Clinician-Rated DT gradually underestimated distress, a tendency towards a greater subjectivity already observed in palliative care in the assessment of prognosis. In a study of 504 patients, Christakis and Lamont [15] showed that physicians were more likely to make mistaken assessments if they knew their patients. In a literature review of cancer prognosis, Vigano [16] observed that physicians tended to overestimate duration of life when they were emotionally involved with their patients.

The Lin’s coefficient concordance was also better than Kappa, which uses a significance threshold of 3/10. If a patient records a distress score of 3/10 and a health professional one of 2/10 then the concordance will be poor even though the difference in value is not very great.

The Clinician-Rated Distress Thermometer was more reliable when performed by psychologists and, to a lesser degree, by nurses. The physicians were the ones who made most of the mistakes, often by underestimating patients’ distress. Is it because they have too medical a representation of distress, based on standard notions of anxiety and depression? This would explain why they did not identify suffering in patients who met neither of the conditions. Further studies are needed on the differences between health professionals in their assessment practice to determine whether other factors are involved such as a closer interpersonal relationship in the caring process, age and experience.

It emerged from our study that past experience can play a role in the ability to recognise when patients are in distress. The psychologists did not challenge the personal interview approach to assessment probably because their training had prepared them to enter more easily into a transferential relationship and to be alert to what patients do not formally express. It is important to be able to identify distress in order to provide relational support and not just treatment by drugs. The definition of Jimmie Holland emphasizes the fact that distress is not a psychiatric symptom but the manifestation of mental adjustment to a terminal illness.

Clinical implications: The performance of the DT as a clinician-reported outcome assessment is reliable at the outset of patient care. Paradoxically, the more we know a patient, the more likely we are to go wrong. Thereafter its use will probably be influenced by the subjectivity of the health professional involved. The DT could be used on patient admission.

## Conclusion

A self-reported Distress Thermometer require that health professional discuss with patients about their distress. Results of this study demonstrate that a subjective feeling like distress can be externally evaluable. When used as ClinRO, DT has a good validity at the beginning of palliative support. The implication for the practice is to allow the health professional to have an evaluation of the distress of the patients even when a discussion is not possible. It is important to be able to identify distress in order to provide relational support rather than a simple drug therapy. The definition of Jimmie Holland emphasizes the fact that distress is not a psychiatric symptom but the manifestation of mental adjustment to a terminal illness. Distress is a universal experience which should not require medical treatment but should benefit from relational support. It is part of the recommendations of NCCN guidelines. The DT is useful at the outset of multidisciplinary patient care. These evaluation need to adapt the management by offering such therapeutic adjustments (psychologist, counseling, treatment ...).

It emerged from our study that past experience can play a role in the ability to recognize when patients are in distress. Future prospects: the quality of clinician-Rated Distress Thermometer should be evaluated taking into account the age of health professionals, their experience in palliative care and their training.

We must now assess whether the training, experience and personality of a professional significantly influence the results of the DT.

## Supplementary information

**Additional file 1.** BDD-NUANCE-V4F430606.accdb. Description of data: data from all patients (demographic and clinical characteristics, DT). (ACCDB 1344 kb)

## Data Availability

All data generated or analysed during this study are included in this published article and its Additional file 1.

## Abbreviations

COD: Cancer outpatient department
DT: Distress Thermometer
HADS: Hospital Anxiety and Depression Scale
IQR: Interquartile range
IRB: Institutional review board
NCCN: National Comprehensive Cancer Network
PCU: Palliative care unit
WHO: World Health Organization
